# Infective SARS-CoV-2 in Skull Sawdust at Autopsy, Finland

**DOI:** 10.3201/eid3008.240145

**Published:** 2024-08

**Authors:** Jonas N. Kantonen, Suvi Kuivanen, Teemu Smura, Henri Puttonen, Eliisa Kekäläinen, Antti Sajantila, Liisa Myllykangas, Anu Kantele, Olli Vapalahti, Mikko I. Mäyränpää, Olli Carpén

**Affiliations:** University of Helsinki, Helsinki, Finland (J.N. Kantonen, S. Kuivanen, T. Smura, H. Puttonen, E. Kekäläinen, A. Sajantila, L. Myllykangas, A. Kantele, O. Vapalahti, M.I. Mäyränpää, O. Carpén);; Helsinki University Central Hospital, Helsinki (J.N. Kantonen, H. Puttonen, E. Kekäläinen, L. Myllykangas, A. Kantele, O. Vapalahti, M.I. Mäyränpää, O. Carpén);; Finnish Institute for Health and Welfare, Helsinki (A. Sajantila)

**Keywords:** COVID-19, SARS-CoV-2, respiratory infections, viruses, zoonoses, tissue distribution, respiratory aerosols and droplets, skull, autopsy, occupational exposure, RT-PCR, viral culture, Finland

## Abstract

We assessed the distribution of SARS-CoV-2 at autopsy in 22 deceased persons with confirmed COVID-19. SARS-CoV-2 was found by PCR (2/22, 9.1%) and by culture (1/22, 4.5%) in skull sawdust, suggesting that live virus is present in tissues postmortem, including bone. Occupational exposure risk is low with appropriate personal protective equipment.

Autopsies afford simultaneous access to all tissues and body compartments. The unique opportunity for extensive sampling during autopsy enables several research questions to be addressed. Early in the COVID-19 pandemic, autopsies were rare, mainly because of presumed transmission risk and shortage of personal protective equipment (PPE), and suspicions that autopsies might be of limited value (*1*,*2*).

Autopsies pose an occupational infectious hazard to the personnel involved in a pathogen-dependent manner. For example, *Mycobacterium tuberculosis* deserves particular attention as a major cause of airborne infections in autopsies that puts pathologists at a 100–200-fold risk for infection compared with the general public (*3*). Viable SARS-CoV-2 has been detected in tissues for prolonged periods after death from COVID-19 (*4*). However, to our knowledge, no confirmed occupational cases of COVID-19 transmitted at autopsies have been reported.

Protection against aerosols remains a challenge in autopsy settings. Bone sawing is a major source of aerosols that can carry pathogens. Sawing of the skull is a standard procedure in every routine autopsy to enable access to the brain. SARS-CoV-2 has previously been documented in bone tissues in 2 reported cases, neither of which were in the skull (*5*). Here, we present results of SARS-CoV-2 analyses from 22 deceased persons with PCR-confirmed COVID-19 and detail our experience of managing the occupational hazards associated with COVID-19 autopsies.

Our study belongs to the Clin_COVID-19 master study approved by the Helsinki University Hospital Ethics committee (approval no. HUS/1238/2020). All autopsies were clinical (nonforensic) and conducted in compliance with research laws and regulations in Finland, after consent from the next of kin.

The postmortem examinations were conducted in the pathology department of the HUS Diagnostic Center in Meilahti, Helsinki, Finland. The series comprised 22 PCR-confirmed cases (any positive airway sample from nasopharynx, bronchi, lungs, tonsils, sclera, or airway-associated cervical or parabronchial lymph nodes) of SARS-CoV-2 identified during 2021–2022 that had skull sawdust sampled during autopsy. Testing was carried out in the pathology and virology laboratories by using accredited and previously published methods (*6*) (Appendix). All autopsies encompassed a neuropathological examination and a collection of swabs/fresh tissues from airway, nonairway, and central nervous system (CNS) categories. In addition, swab samples were collected from skull sawdust and the contaminated autopsy table with the organ block. Each tissue was sampled with separate sterile equipment. PCR-positive samples were cultured using VeroE6 cells to assess for infective SARS-CoV-2. 

We detected SARS-CoV-2 by reverse transcription PCR in 22/22 (100%) airway, 10/22 (45.5%) nonairway, 0/22 CNS, 2/22 (9.1%) skull sawdust, and 13/22 (59.1%) autopsy table samples (Table). The virus was culturable in 13/22 (59.1%) airway, 2/22 (9.1%) nonairway, 1/22 (4.5%) skull sawdust, and 3/22 (13.6%) autopsy table samples.

**Table:** SARS-CoV-2 distribution among cohort of 22 autopsied deceased persons with COVID-19 who had skull sawdust sampling, Finland*

Case no.	Airway			Nonairway		CNS PCR and culture	Skull sawdust			Autopsy table	
	PCR	Culture		PCR	Culture		PCR	Culture		PCR	Culture
1	+	–		–	–	–	–	–		+	–
2	+	–		–	–	–	–	–		–	–
3	+	–		–	–	–	–	–		–	–
4	+	–		–	–	–	–	–		–	–
5	+	–		–	–	–	–	–		–	–
6	+	+		–	–	–	–	–		+	–
7	+	+		+	–	–	–	–		+	–
8	+	+		+	–	–	–	–		–	–
9†	+	+		+	–	–	+	+		+	–
10	+	+		–	–	–	–	–		+	–
11	+	+		+	+	–	–	–		+	–
12	+	+		–	–	–	+	–		–	–
13	+	–		–	–	–	–	–		–	–
14	+	+		+	+	–	–	–		+	–
15	+	+		+	–	–	–	–		+	+
16	+	+		+	–	–	–	–		+	+
17	+	–		–	–	–	–	–		+	–
18	+	+		+	–	–	–	–		+	–
19	+	–		+	–	–	–	–		+	–
20	+	+		+	–	–	–	–		–	–
21	+	–		–	–	–	–	–		–	–
22	+	+		–	–	–	–	–		+	+
Positive samples/total no. samples (%)	22/22 (100)	13/22 (59.1)		10/22 (45.5)	2/22 (9.1)	0/22	2/22 (9.1)	1/22 (4.5)		13/22 (59.1)	3/22 (13.6)

*The pooled sample category per patient was considered positive if a single positive tissue sample of that category was found (copy number cutoff value 10; Appendix). Airway refers to tissues relating to the airway system (i.e., nasopharynx, bronchi, lungs, tonsils, sclera, and airway-associated cervical and parabronchial lymph nodes). Autopsy table refers to the contaminated autopsy table and the outer surfaces of the organ block, representing the main working area and target of showering with water. Only a limited number of cases showed culture positivity (Ct value reduction after culture compared with initial Ct value; Appendix) in general; skull positivity was scarce, whereas the autopsy table was more often positive by both PCR and viral culture. †Test results showed culture positivity in the cranial sawdust sample and low-level nonairway PCR-positivity limited to skeletal muscle and salivary gland tissues, indicating limited systemic viral involvement (Appendix Table).

Among the personnel present at COVID-19 autopsy procedures, no cases of COVID-19 resulting from occupational exposure were identified. Serologic screening results of all persons involved in COVID-19 autopsies (n = 5) in June 2020 were negative, and none showed PCR positivity when tested during symptoms.

Our findings revealed that SARS-CoV-2 was detectable by PCR in 9.1% and by viral culture in 4.5% of skull sawdust samples, suggesting the presence of live virus and a risk, although low, of infective viruses becoming aerosolized. We could not identify previous work examining cranial sawdust for the presence of pathogens, but our results align with a previous study showing SARS-CoV-2 PCR positivity for 4.5% of goggles and no masks tested after autopsy (*7*).

The sample size for our study was limited but represents a consecutive and nonselected series of cases at a single institution. We did not directly assess aerosols, but given that bone sawing is the only high-energy technique used, and considering the findings from a previous study (*7*), the presence of concomitant other sources of infective aerosols in the autopsy room is unlikely. The personnel present during COVID-19 autopsies were not systematically tested, but symptomatic persons were extensively PCR tested for SARS-CoV-2 during the study period (2020–2022). In addition, skull sawdust samples might not consist solely of bone and could contain adjacent tissues because of anatomy, particularly the frontal sinus, which is lined with respiratory epithelium. Skullcap sawing has the potential to generate infective aerosols, but in our experience, general autopsy safety measures are effective. The absence of positive findings in our CNS samples give confidence in the sterility of our sampling technique, thereby making other sources of contamination in the skull sawdust samples less likely.

Pandemic preparedness should encompass plans for early, rapid autopsies to acquire vital data at the onset. General safety measures appear adequate for most pathogens encountered during autopsy, including SARS-CoV-2 (*3*). However, early testing for pathogens in skull sawdust, along with other tissues, could prove beneficial in further assessing the risk for occupational infections resulting from autopsies during future pandemics.

Appendix Additional information on infective SARS-CoV-2 in skull sawdust at autopsy, Finland.

## References

[1] Ledford H. Autopsy slowdown hinders quest to determine how coronavirus kills. Nature. 2020. 10.1038/d41586-020-01355-z 32382121

[2] Fineschi V, Aprile A, Aquila I, Arcangeli M, Asmundo A, Bacci M, et al.; Scientific Society of Hospital Legal Medicine of the National Health System (COMLAS); Italian Society of Anatomical Pathology and Cytology (SIAPEC). Management of the corpse with suspect, probable or confirmed COVID-19 respiratory infection - Italian interim recommendations for personnel potentially exposed to material from corpses, including body fluids, in morgue structures and during autopsy practice. Pathologica. 2020;112:64–77. 32324727 10.32074/1591-951X-13-20 PMC7931563

[3] Kritselis M, Remick DG. Universal precautions provide appropriate protection during autopsies of patients with infectious diseases. Am J Pathol. 2020;190:2180–4. 10.1016/j.ajpath.2020.08.005 32827462 PMC7437536

[4] Plenzig S, Bojkova D, Held H, Berger A, Holz F, Cinatl J, et al. Infectivity of deceased COVID-19 patients. Int J Legal Med. 2021;135:2055–60. 10.1007/s00414-021-02546-7 33665704 PMC7932833

[5] Jurek T, Rorat M, Szleszkowski Ł, Tokarski M, Pielka I, Małodobra-Mazur M. SARS-CoV-2 viral RNA is detected in the bone marrow in post-mortem samples using RT-LAMP. Diagnostics (Basel). 2022;12:515. 10.3390/diagnostics12020515 35204605 PMC8870857

[6] Corman VM, Landt O, Kaiser M, Molenkamp R, Meijer A, Chu DK, et al. Detection of 2019 novel coronavirus (2019-nCoV) by real-time RT-PCR. Euro Surveill. 2020;25:2000045. 10.2807/1560-7917.ES.2020.25.3.2000045 31992387 PMC6988269

[7] Brandner JM, Boor P, Borcherding L, Edler C, Gerber S, Heinemann A, et al. Contamination of personal protective equipment during COVID-19 autopsies. Virchows Arch. 2022;480:519–28. 10.1007/s00428-021-03263-7 34993593 PMC8735722

