# Supplementary material for: Infective SARS-CoV-2 in Skull Sawdust at Autopsy, Finland
Source: Emerg Infect Dis. 2024 Aug;30(8):1735–7. doi: 10.3201/eid3008.240145 (PMC11286058; doi:10.3201/eid3008.240145)
Supplement: Appendix — Additional information on infective SARS-CoV-2 in skull sawdust at autopsy, Finland. [file 24-0145-Techapp-s1.pdf]

Article DOI: https://doi.org/10.3201/eid3008.240145

EID cannot ensure accessibility for supplementary materials supplied by authors. Readers who have difficulty accessing supplementary content should contact the authors for assistance.

Infective SARS-CoV-2 in Skull Sawdust at Autopsy, Finland

Appendix

Autopsy facilities and practices

The autopsy facilities at our department include three autopsy suites. All three have negative air pressure, a minimum of 15 air changes per hour, with one suite dedicated to infectious diseases. The dedicated room uses a HEPA-filter for the exhaust air, and all autopsy suites offer a local HEPA-filtered air purifying unit for aerosol control. Two of the COVID-19 cases were done in routine autopsy suites due to unknown infection status, but these cases did not show skull positivity. All the identified COVID-19 cases were autopsied in the dedicated autopsy suite for infectious cases.

The autopsy protocol used en bloc removal of the viscera, and all autopsies included assessment of the central nervous system via craniotomy. The skull was opened using an oscillating saw, under the hood of the local air purifying unit for aerosol control. Cold water was used sparingly, with minimal pressure to avoid aerosol formation.

The personnel involved in the COVID-19 autopsies included five autopsy technicians and four pathologists. One to two pathologists and autopsy technicians were present in the autopsy suite for a single case. The duration of autopsies varied from 1–4 hours per case.

Personal protective equipment (PPE) used routinely consisted of an FFP3 mask, cap, goggles, water resistant gown, double pairs of gloves and boots. A powered air purifying respirator (VersaFlo, 3M) was used in some cases instead of the FFP3 mask and goggles.

SARS-CoV2 tissue processing, virus isolation and quantitative RT-PCR

For SARS-CoV2+ cases fresh tissue samples, swabs (including swabs of skull sawdust and swabs of the contaminated autopsy table with the organ block), cerebrospinal fluid (CSF) and blood were collected at autopsy and transferred to the biosafety level 3 laboratory (BSL-3) for further processing. For virus isolation, tissue samples were homogenized using mortar and pestle and sterile sand with ice-cold PBS. Cleared tissue samples were collected, inoculated on Vero E6 cells, and incubated for 1 h at +37°C 5% CO2, after which the cells were washed with PBS and virus growth media containing 2% FCS was added. Virus growth was followed by cytopathic effect (CPE) formation and confirmed by quantitative RT-PCR on day 0 and between days 4–11, with >2-log value increase considered positive. See Appendix Table for culture positive samples per sample category.

RNA was extracted from the tissue samples using TRIzol reagent (Invitrogen) according to the manufacturer's instructions, and from cell culture supernatants using the QIAamp Viral RNA Mini Kit (Qiagen). Quantitative SARS-CoV-2 RT-PCR was performed according to previously published protocol (*1*). See Appendix Table for the tissue with highest SARS-CoV-2 copy number per category.

High SARS-CoV-2 copy numbers correlated with positive viral culture (rank biserial correlation for airway samples $r_{rb} = 0.783$ (n=22) and all cultured samples $r_{rb} = 0.646$ (n=47), both $p < 0.01$), as reported in other studies (*2*,*3*). The data in this cohort does not allow for reliable evaluation of the effect of postmortem delay on culture positivity. However, our experience is similar to the findings seen by others, with surprisingly long postmortem delays showing positivity (*4*).

Serological testing

The autopsy personnel were tested for presence of antibodies after the first wave of infections in June 2020. The tested cohort included all 6 individuals that had known exposure to COVID-19 autopsies at the time of testing. None of the individuals either had symptomatic disease warranting PCR-testing, or their PCR-tests were negative in the time prior to antibody testing. A total of five COVID-19 autopsies were performed during the spring of 2020, with these cases not being part of the cohort reported on here.

Experience on occupational hazards

In our experience FFP3 masks as part of aerosol controlling autopsy precautions seem to protect from airborne infections during autopsy. No cases of autopsy-related COVID-19 were identified among the personnel in Helsinki during the years 2020 – 2023. In addition, no cases of TB have been identified while using the same safety procedures during the years 2011 – 2023. The addition of local exhausts as a source-control with HEPA-filters helps further reduce the amount of potentially infective aerosols in the autopsy room, easing the workload of masks as the single method for aerosol filtering.

References

1. Corman VM, Landt O, Kaiser M, Molenkamp R, Meijer A, Chu DK, et al. Detection of 2019 novel coronavirus (2019-nCoV) by real-time RT-PCR. Euro Surveill. 2020;25:2000045. PubMed https://doi.org/10.2807/1560-7917.ES.2020.25.3.2000045

2. Huang CG, Lee KM, Hsiao MJ, Yang SL, Huang PN, Gong YN, et al. Culture-based virus isolation to evaluate potential infectivity of clinical specimens tested for COVID-19. J Clin Microbiol. 2020;58:e01068–20. PubMed https://doi.org/10.1128/JCM.01068-20

3. Berg MG, Zhen W, Lucic D, Degli-Angeli EJ, Anderson M, Forberg K, et al. Development of the RealTime SARS-CoV-2 quantitative Laboratory Developed Test and correlation with viral culture as a measure of infectivity. J Clin Virol. 2021;143:104945. PubMed https://doi.org/10.1016/j.jcv.2021.104945

4. Plenzig S, Bojkova D, Held H, Berger A, Holz F, Cinatl J, et al. Infectivity of deceased COVID-19 patients. Int J Legal Med. 2021;135:2055–60. PubMed https://doi.org/10.1007/s00414-021-02546-7

Appendix Table. Highest SARS-CoV-2 copy number values per sample category, with culture positive tissue type where applicable, ranked according to PCR copy number*

Case no.	Airway PCR	Airway culture	Non-airway PCR	Non-airway culture	CNS PCR & culture	Skull PCR	Skull culture	Table PCR	Table culture
1	493000 (lung)	-	-	-	-	-	-	1090 (table)	-
2	27570 (tonsil)	-	-	-	-	-	-	-	-
3	1114000 (lung)	-	-	-	-	-	-	-	-
4	41170 (tonsil)	-	-	-	-	-	-	-	-
5	176100 (tonsil)	-	-	-	-	-	-	-	-
6	111400 (bronchi)	bronchi, lung, tonsil	-	-	-	-	-	6840 (table)	-
7	18180000 (lung)	lung, tonsil, nasopharynx	15260 (heart)	-	-	-	-	206 (table)	-
8	15000000 (cervical lymph node)	cervical lymph node	1028 (pancreas)	-	-	-	-	-	-
9	5476000 (nasopharynx)	nasopharynx, bronchi, tonsil	8465 (salivary gland)	-	-	74830 (skull)	skull	36.87 (table)	-
10	3552000 (nasopharynx)	bronchi	-	-	-	-	-	8625 (table)	-
11	7929000 (bronchi)	bronchi	182700 (gut)	gut	-	-	-	1201 (table)	-
12	1504000 (nasopharynx)	nasopharynx	-	-	-	53360 (skull)	-	-	-
13	3006 (parabronchial lymph node)	-	-	-	-	-	-	-	-
14	365400 (bronchi)	nasopharynx, sclera, parabronchial lymph node	13980 (heart)	muscle	-	-	-	10360 (table)	-
15	134500000 (lung)	lung	1249 (salivary gland)	-	-	-	-	9470 (table)	table
16	44870000 (lung)	lung, bronchi	14810 (spleen)	-	-	-	-	3492 (table)	table
17	455200 (lung)	-	-	-	-	-	-	32387 (table)	-
18	2486000 (nasopharynx)	lung	135.3 (salivary gland)	-	-	-	-	13550 (table)	-
19	591700 (nasopharynx)	-	828.5 (thyroid)	-	-	-	-	33320 (table)	-
20	6553000 (lung)	lung, tonsil	21480 (salivary gland)	-	-	-	-	-	-
21	24200 (parabronchial lymph node)	-	-	-	-	-	-	-	-
22	917600 (nasopharynx)	nasopharynx, bronchi	-	-	-	-	-	23120 (table)	table

*The airway samples showed systematically higher copy numbers of SARS-CoV-2 when compared to other sample categories. Positive skull samples were found in cases showing high copy numbers in the nasopharynx. For statistical testing the copy number data were ranked, and culture was only deemed positive if the sample showing the highest copy number showed culture positivity. Using rank biserial correlation we obtained correlation coefficients r_{rb} = 0.783 (n=22, p <0.01) for airway samples and r_{rb} = 0.646 (n=47, p <0.01) for all cultured sample categories, showing correlation between high copy number and viral culture positivity. Airway refers to tissues relating to the airway system (i.e. nasopharynx, bronchi, lungs, tonsils, sclera, and airway-associated cervical and parabronchial lymph nodes). Skull refers to skull sawdust. Table refers to the contaminated autopsy table and the outer surfaces of the organ block, representing the main working area and target of showering with water.
